# Novel enzymes involved in the biotransformation of oxytetracycline by *Arthrobacter nicotianae* OTC-16

**DOI:** 10.1128/spectrum.00562-25

**Published:** 2025-08-12

**Authors:** Beibei Wang, Weijie Xu, Keke Wang, Hui Lin, Xin Zhang, Zulfiqar Ahmad

**Affiliations:** 1 College of Forest and Biotechnology, Zhejiang A & F University, Hangzhou, China; 2 Institute of Environment, Resource, Soil and Fertilizer, Zhejiang Academy of Agricultural Sciences, Hangzhou, PR China; 3 Laboratoire Ondes et Milieux complexes (LOMC), UMR 6294 CNRS, University of Le Havre Normandy (ULHN), Le Havre, France; DePauw University, Greencastle, Indiana, USA

**Keywords:** oxytetracycline, transcriptomics, proteomics, degradation mechanisms, biodegradation enzymes

## Abstract

Current research on the microbial degradation of antibiotic residues primarily focuses on isolating degrading bacteria and characterizing their metabolites. The enzymatic mechanisms underlying these biotransformation processes remain poorly understood. Here, we investigated the molecular mechanisms involved in the biodegradation of oxytetracycline (OTC) by *Arthrobacter nicotianae* OTC-16 using integrated transcriptomic and proteomic sequencing analyses. OTC exposure significantly altered gene expression, identifying 1,158 and 863 differentially expressed genes on days 2 and 4, respectively. Proteomics revealed 352 and 818 differentially expressed proteins, predominantly involved in compound binding and redox processes. Integrated analysis identified *AlkB* (DNA oxidative demethylase), *uaZ* (urate oxidase), and *prpD* (2-methylcitrate dehydratase) as key enzymes mediating OTC biodegradation through decarboxylation, carbon-carbon bond reduction, and dehydration reactions, confirmed by RT-qPCR. Protein-protein interactions subsequently highlighted supportive proteins, including glutathione S-transferase, ABC transporter ATP-binding protein, prpB, prpE, MarR regulators, and glyoxalase. Notably, *prpD* and *uaZ* were successfully expressed in *Escherichia coli*, and their OTC degradation-promoting activities were validated through *in vitro* assays. This study is the first to report the roles of *prpD* and *uaZ* in antibiotic residue degradation, providing novel insights into the enzymatic mechanisms and laying a foundation for future genetic engineering and practical applications.

**IMPORTANCE:**

Oxytetracycline (OTC), a widely used antibiotic in agriculture and animal husbandry, frequently accumulates in the environment, posing significant ecological and human health risks by promoting antibiotic resistance. Understanding the microbial enzymatic pathways for OTC biodegradation is crucial for developing effective bioremediation strategies. This study provides a comprehensive molecular analysis of the OTC degradation process by *Arthrobacter nicotianae* OTC-16, identifying three key enzymes (*AlkB*, *uaZ*, and *prpD*) involved in OTC removal. Successfully expressing *prpD* and *uaZ* in *Escherichia coli* and validating their biodegradation activity *in vivo* further underscores their potential for biotechnological applications. These findings significantly enhance the knowledge of microbial antibiotic degradation mechanisms, offering practical molecular targets for environmental detoxification strategies.

## INTRODUCTION

Tetracyclines (TCs), such as tetracycline, chlortetracycline, oxytetracycline (OTC), and doxycycline, are among the most widely used antibiotics worldwide (1). The overuse and release of antibiotics into the environment without treatment pose a great threat to the ecosystem and public health (2). Microbial bioremediation has emerged as an effective approach for removing antibiotic residues from the environment, as it is cost-effective, is efficient, and does not produce secondary pollution (3–5). Currently, most research on antibiotic biodegradation focuses on screening efficient degrading microorganisms, characterizing degrading pathways, and analyzing metabolic processes. TC-degrading bacteria isolated to date include OTC-degrading bacteria, such as *Bacillus brevis* MM2 (3), *Pseudomonas aeruginosa* OTC-T (6), *Mycolicibacterium* sp. (7), and *Arthrobacter nicotianae* OTC-16 (8); TC-degrading bacteria, such as *Sphingobacterium* sp. WM1 (9), *Klebsiella* sp. SQY5 (10), and *Stenotrophomonas maltophilia* DT1 (11); doxycycline-degrading bacteria, such as *Brevundimonas naejangsanensis* DD1 and *Sphingobacterium mizutaii* DD2 (12); and chlortetracycline-degrading bacteria, such as *Bacillus cereus* LZ01 (13). The metabolic pathways of some TCs have been elucidated, including TCs (14–16) and OTCs (8, 17). However, the enzymatic systems and metabolic pathways underlying microbial biotransformation remain poorly understood. The biotransformation pathways of TC antibiotics mainly include demethylation, deamination, decarbonylation, hydroxylation, enolone isomerization, epimerization, and ring opening, and many studies have proposed that monooxygenases, dehydrogenases, dioxygenases, peroxidases, oxidoreductases, superoxide dismutases, and acyltransferases may be involved in these biotransformation processes (2, 10, 14–16, 18). Among them, oxidases and oxidoreductases hydroxylate aromatic compounds with active molecular oxygen and cleave ring structures (19, 20). Superoxide dismutase promotes protein degradation through methylation (11). Dehydrogenases and transferases inactivate TC antibiotics by eliminating sensitive chemical bonds (15). However, most of the above conclusions based on multi-omics analyses of genomes and transcriptomes of the strains and the enzymatic properties of proteins are speculative. To date, few studies have investigated the specific sites and roles of these enzymes in the degradation of TC antibiotics.

A previous study isolated the efficient OTC-degrading bacterium *Arthrobacter nicotianae* OTC-16, which degraded 91.54% of OTC residues, 55.81% of TC, and 22.63% of chlortetracycline in aqueous media (8), indicating its potential for development and utilization. This strain can biotransform and detoxify OTC through decarbonylation of ring A, reduction of C = C bonds by the addition of two hydride ions, and dehydration of ring C, sequentially (8). However, the molecular mechanism and degrading enzyme systems underlying the biodegradation process remain unclear.

To our knowledge, strain OTC-16 has the highest OTC degradation efficiency among strains identified to date. Moreover, most of the antibiotic-degrading bacteria isolated in the past belong to the phylum Proteobacteria. The strain obtained in this study belongs to the phylum Actinobacteria, which includes bacteria with strong stress resistance, such as tolerance to high temperatures (21), and thus would exhibit strong environmental adaptation. Based on its unique genetic background and favorable degradation characteristics, strain OTC-16 likely has a unique degrading enzyme system that differs from that of other degrading bacteria. Therefore, based on our previous research on the intrinsic mechanism underlying the strain’s multiple antibiotic resistance (22), the present study aimed to explore the molecular mechanisms underlying OTC degradation by strain OTC-16 via transcriptome and proteome sequencing analyses conducted after 2 and 4 days of OTC exposure and identify the key enzyme genes and related proteins involved in the degradation process. This study is significant because it provides a better understanding of the biodegradation mechanism of OTC and a theoretical basis and molecular components for genetically modifying and applying the strain.

## MATERIALS AND METHODS

### Strain culture

To study the gene expression of the OTC-degrading strain OTC-16 under OTC stress, 50 µL of strain OTC-16 at an initial concentration of 10^8^ cfu/mL was inoculated into test tubes containing 5 mL Luria-Bertani (LB) broth spiked with OTC at a concentration of 100 mg/L, which has been confirmed to be the optimal initial concentration for the degradation of the strain (8). The test tubes were then cultured at 28°C for 4 days (T). Bacterial suspensions were sampled on days 2 and 4 post-incubation, and the collected cells were used for transcriptome and proteome analyses. The experiments were performed in triplicate, and the cells cultured in LB broth without OTC were used as the control (CK) to assess gene expression differences.

### RNA sequencing and analysis

Total RNA was extracted from cells cultured in LB broth with and without OTC (100 mg/L) on the second and fourth days according to the method of Li (23). Total RNA was reverse transcribed into cDNA to construct a cDNA library. Transcriptome sequencing was performed using combinatorial probe-anchored assembly sequencing (cPAS) technology on a DNBSEQ-G400 sequencing platform (Beijing Genomics Institute, China). Raw reads were filtered to remove low-quality reads containing at least three ambiguous nucleotides and a q-value <20, and the filtered reads were aligned to the reference genome sequence (NZ_CP033081) using Bowtie2 (24). The gene expression values for each sample were calculated and normalized using the fragments per kilobase per million (FPKM) method and then analyzed using DESeq (version 1.18.0), with the conditions for differentially expressed genes (DEGs) set at |log2-fold change| > 1 and *P*-value < 0.05. Gene Ontology (GO) and Kyoto Encyclopedia of Genes and Genomes (KEGG) annotations of the DEGs were conducted using Goseq and KOBAS software, respectively.

### Proteome sequencing and analysis

Suspensions of strain OTC-16 cultured in LB broth with and without OTC (100 mg/L) were collected on days 2 and 4. After treatment with a protein extraction reagent (Beyotime Biotechnology, Shanghai, China) at room temperature for 15 min, the sample solution was centrifuged at 12,000 rpm for 10 min to collect the supernatant. The supernatant was transferred to a fresh tube, and the protein quality was determined using sodium dodecyl sulfate-polyacrylamide gel electrophoresis (SDS-PAGE). Proteins were quantified according to the method described by Chen (25).

Raw data from data-independent acquisition (DIA) data were processed and analyzed using isobaric tags for relative and absolute quantitation (SCIEX, United States) with the default parameters. Then, the data were imported directly into the Proteome Discoverer 2.2 software for a database search. To improve the quality of the analysis, the search results were further filtered using Proteome Discoverer 2.2 software. Peptide spectrum matches with more than 99% confidence were designated as trusted PSMs, and proteins containing at least one unique peptide were considered trusted proteins. Finally, false discovery rate (FDR) verification was performed, and peptides and proteins with an FDR greater than 1% were removed. The proteins were then annotated using the protein family alignment database and the Hidden Markov Model (Pfam) database (26). Differentially expressed proteins (DEPs) were filtered according to Chen (25). The protein-protein interaction (PPI) network was visualized using Cytoscape version 3.9.1 (27).

### Validation of degradation genes

#### RT-qPCR

RT-qPCR was performed to verify the expression levels of screened genes. Total RNA was extracted from each sample using the TRIzol method (23), and then, 1 µg of RNA was used to synthesize cDNA using the Prime Script TMRT Reagent Kit (TaKaRa, Dalian, China). The primer sequences for the three screened genes and the reference gene *16S rRNA* are listed in Table 1. RT-qPCR was performed using a BIOER real-time fluorescence quantitative PCR instrument (FQD-96C, Hangzhou). The reactions were conducted in a 10 µL volume containing 1 µL DNA template, 5 µL SYBR Green PCR Supermix, 2.8 µL primers, and 1.2 µL sterile water. RT-qPCR was performed under the following conditions: 95°C for 5 min, followed by 45 cycles of 95°C for 15 s, 60°C for 30 s, and 72°C for 30 s. The relative expression levels of the target genes were determined using the standard curve method described by Xu (28).

**TABLE 1:** Primer sequences used for RT-qPCR amplification in the study

Primer name	Primer sequence (5’−3’）
*prpD*-F	GATCATCAACCGCATCATC
*prpD*-R	GAAGGTGTCGTGGTAGTC
*AlkB*-F	AGGAATACCAGCCTGATG
*AlkB*-R	GAATCGCCAATGGACAATG
*uaZ*-F	TCCTTCAGTGTCGTGTAC
*uaZ*-R	CTTCTCGCAGAACAAGTC
*16sr*RNA-F	CCTACGGGAGGCAGCAG
*16sr*RNA-R	ATTACCGCGGCTGCTGG

#### Construction of recombinant expression vectors

To validate the activity of the degradation-related genes, they were cloned and expressed *in vitro*. The plasmid pET-28a (+) (Takara, Beijing, China), which allows cloning and expression in *E. coli*, was used as the expression vector. The plasmid contained a kanamycin resistance marker as well as *BamHI*, *HindIII*, and *EcoRI* restriction enzyme sites, the details of which are shown in Fig. S1A.

DNA was extracted using a Bacterial Genomic DNA Kit (TaKaRa, Dalian, China) and amplified via PCR using the primers listed in Table 2 to obtain the target fragments of genes *prpD*, *uaZ*, and *AlkB*. The genes *prpD* and *uaZ* were double-digested with the restriction enzymes *BamHI* and *HindIII*, whereas *AlkB* was double-digested with *EcoRI* and *HindIII*. The resulting fragments were then ligated into plasmid pET-28a (+) through enzymatic digestion, respectively. Subsequently, these recombinant plasmids were transformed into *E. coli* DH5α (Takara, Beijing, China) for cloning. Successful clones were verified by PCR amplification using the primers listed in Table 2 and subsequent electrophoresis of the amplification products. The amplification products of the expected size were sequenced (Qingke Biotech, Shanghai, China). Plasmids with verified sequences were used for heterologous expression analysis.

**TABLE 2:** PCR amplification primers of degradation genes^*a*^

Primer name	Primer sequence (5′−3′）
pET-*prpD*-F	gagctgtaGGATCCATGATCAACCACCCAGTACGC(*BamHI*)
pET-*prpD-*R	cccAAGCTTTTAGAACAGGCCCTTCGG(*HindIII*)
pET-*uaZ*-F	gagctgtaGGATCCATGACTACCGAAATCACCGCCCCAGCAGAC(*BamHI*)
pET-*uaZ*-R	cccAAGCTTTTAGATAAATGCCGGAATATTTGCCCAGATCG(*HindIII*)
pET-*AlkB*-F	ccgGAATTCATGGAGTCGCTTTTCGATGACAGTGCATTG(*EcoRI*)
pET-*AlkB*-R	cccAAGCTTCTAGACCGCTTCCAGCCCGGTCTGGCGCAG(*HindIII*)

^*a*^ Underlined sequences indicate restriction sites.

#### Heterologous expression of degradation genes

In the study, *E. coli* BL21(DE3) was used as the expression host. The recombinant plasmids pET-28a (+)-*AlkB*, pET-28a (+)-*uaZ*, and pET-28a (+)-*prpD* were transformed into *E. coli* BL21(DE3) competent cells using the method described by Cui (29). The final suspension was inoculated into LB broth spiked with 50 mg/mL kanamycin and cultured until the OD_600_ of the suspension reached 0.5–0.8. Protein expression was induced by adding 0.5 mM isopropyl-β-d-thiogalactopyranoside (IPTG). After overnight induction, proteins were extracted using a protein extraction kit (Beyotime Biotechnology, Shanghai, China). The extracted protein samples were run at 80 V for 30 min and 120 V for 1.5 h on an SDS-PAGE gel (Beyotime Biotechnology, Shanghai, China). The SDS-PAGE gel was stained with 50 mL of Coomassie Brilliant Blue dye (Beyotime Biotechnology, Shanghai, China) and then decolorized. In this study, protein samples from uninduced transformants were used as negative controls, whereas those from the strain containing the pET-28a (+) vector were used as positive controls.

#### Degradation effect of gene products

The degradation effects of the gene products were confirmed using an OTC degradation experiment. The strain OTC-16 was inoculated into 5 mL of LB broth and incubated at 30°C and 180 rpm to OD_600_ = 1.4 to generate the seed solution. Proteins prpD and uaZ were prepared according to the method described in Section Heterologous expression of degradation genes. Degradation experiments were conducted in Erlenmeyer flasks containing 50 mL LB broth spiked with 100 mg/mL OTC.

Seven different treatments were set up for the OTC degradation experiment, including (i) inoculating 1% (vol/vol) strain OTC-16 only; (ii) inoculating 1% (vol/vol) strain OTC-16 and adding 0.1 mg prpD protein; (iii) inoculating 1% (vol/vol) strain OTC-16 and adding 0.1 mg uaZ protein; (iv) inoculating 1% (vol/vol) strain OTC-16 and adding 0.1 mg prpD protein and 0.1 mg uaZ protein; (v) adding 0.1 mg prpD protein; (vi) adding 0.1 mg uaZ protein; and (vii) adding 0.1 mg prpD protein and 0.1 mg uaZ protein. The seven treatments were introduced into Erlenmeyer flasks and adjusted to the same final volume. All Erlenmeyer flasks were then incubated at 30°C and 180 rpm for 4 days. The OTC in the aqueous solutions was determined on days 2 and 4 using high-performance liquid chromatography (HPLC). The culture solution was centrifuged at 12,000 rpm for 5 min, after which the supernatant was extracted, diluted 10-fold with sterilized water, and filtered through a 0.22 µm microporous membrane. The filtrate was then frozen and stored at −20°C for analysis. HPLC analysis of the filtrate was performed as described by Shi (8).

## RESULTS AND DISCUSSION

### RNA-seq analysis of OTC biotransformation

**Fig 1:**
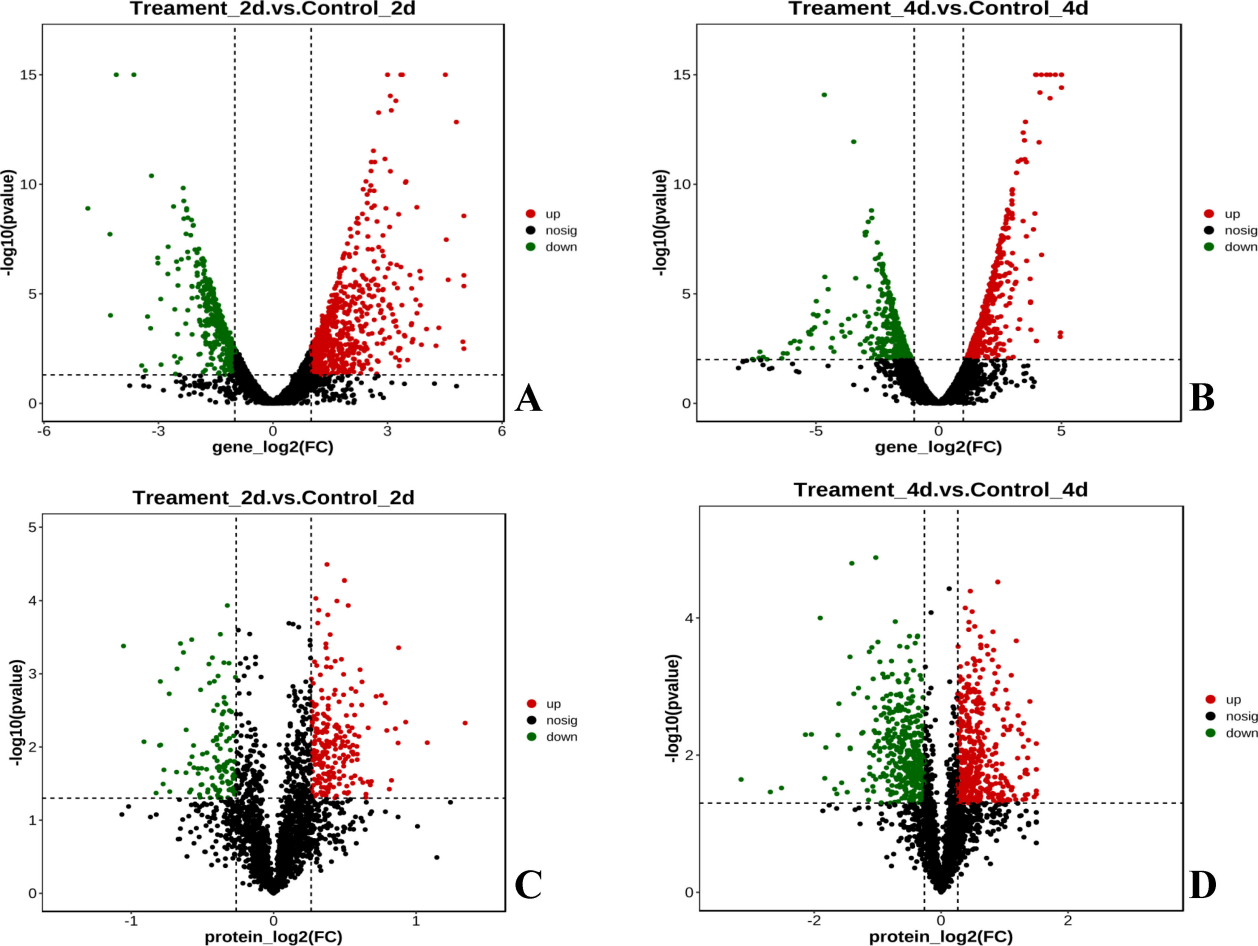
Volcano plots of DEGs and DEPs. (**A**) Volcano plots of DEGs on the 2nd day. (**B**) Volcano plots of DEGs on the 4th day. (**C**) Volcano plots of DEPs on the 2nd day. (**D**) Volcano plots of DEPs on the 4th day. (|log2FoldChange (log2FC)|＞1 and *P*＜0.05).

After 2 and 4 days of OTC exposure, the total RNA of strain OTC-16 from the different treatments was extracted, and transcriptome analysis was performed. The sequencing results showed that the error rate of transcriptome data was ≤0.03%, the percentage of bases with a Q30 score was >93%, and the GC content of each sample ranged from 57.07% to 59.78% (Table S1). As shown in Fig. S2, inter-group differences among the samples were small, intra-group reproducibility was good, and the biological replication index had an R^2^ value >0.8, indicating the reliability of the sequencing results. Genes with |log2FoldChange| > 1 and *P* < 0.05 were selected as DEGs. As shown in Fig. 1, OTC exposure altered the gene expression pattern of strain OTC-16. In total, 1,158 DEGs were identified on the second day of OTC exposure, including 631 upregulated and 527 downregulated genes (Fig. 1A). On day 4, 863 DEGs were identified, including 454 upregulated and 409 downregulated genes (Fig. 1B).

**Fig 2:**
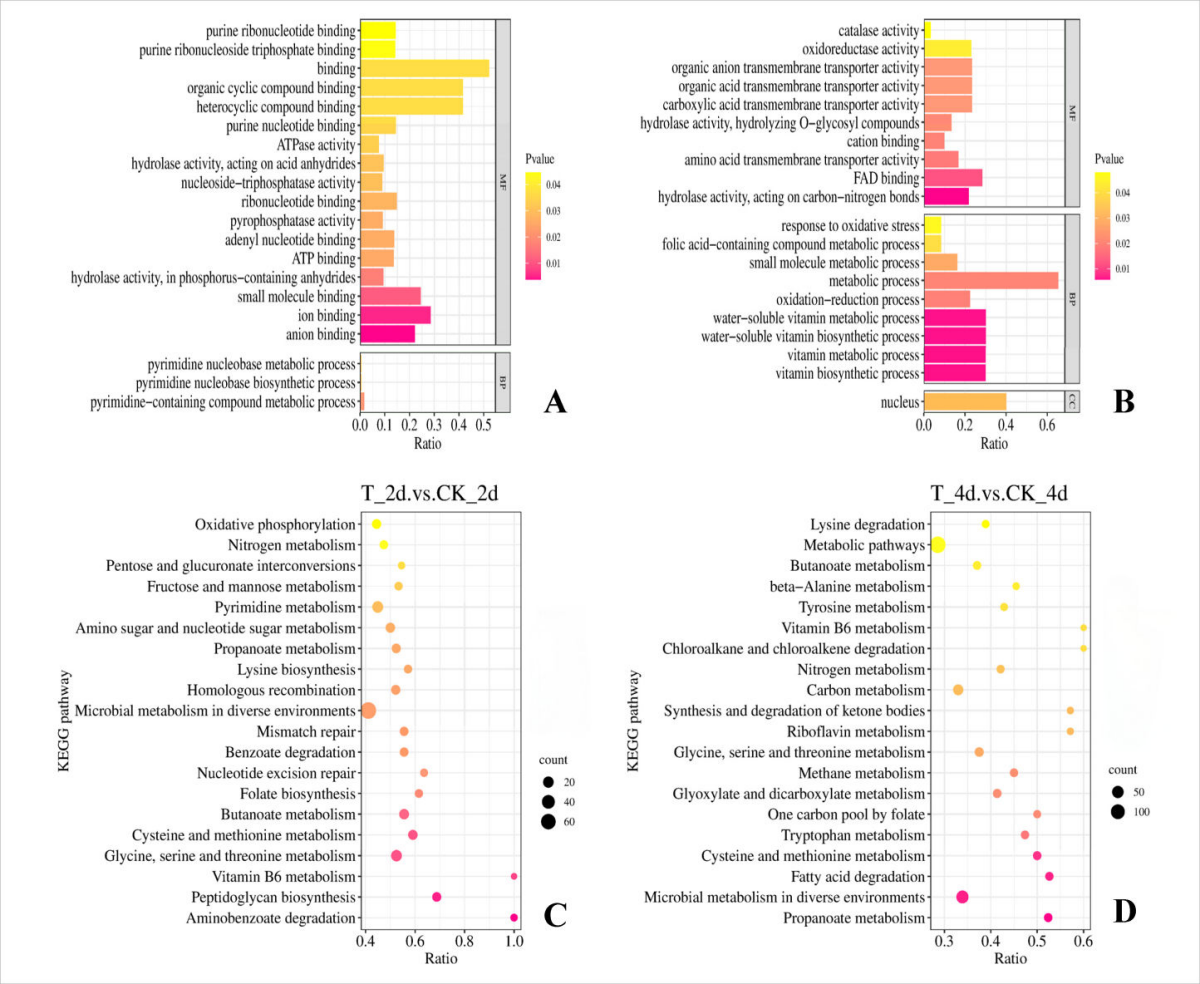
GO enrichment analysis circle plot and KEGG enrichment analysis bubble plot of DEGs of strain OTC-16. (**A**, **B**) GO enrichment analysis circle plot of DEGs on the 2nd and 4th day, respectively; (**C**,** D**) KEGG enrichment analysis bubble plot of DEGs on the 2nd and 4th day, respectively. In the GO enrichment analysis circle plot, the darker the color of the horizontal column, the more significant the difference. In the KEGG enrichment analysis bubble plot, the area of the dots is directly proportional to the enrichment degree of the metabolic pathways, with a larger area of the dots corresponding to a greater intensity of pathway enrichment.

DEGs showing significant differences at the 0.05 level underwent GO enrichment analysis. GO enrichment analysis circle plot (Fig. 2A and B) showed that the metabolic behavior of the strain differed slightly between days 2 and 4. On day 2, the DEGs were primarily enriched in processes related to the binding of cyclic compounds, including organic cyclic compounds and heterocyclic compounds. On day 4, the DEGs were mainly enriched in metabolic processes. Enrichment analysis revealed significant upregulation of pathways related to cyclic compound binding, metabolism, and redox processes in strain OTC-16, including “organic cyclic compound binding” (GO:0097159), “heterocyclic compound binding” (GO:1901363), “heterocyclic compound metabolic process” (GO:0046483), “organic cyclic compound metabolic process” (GO:1901360), and “oxidoreductase activity” (GO:0055114, GO:0016491). Conversely, the analysis revealed significant downregulation of pathways involved in the “synthesis and metabolism of vitamins” (GO:0006766, GO: 0009110) and “purine” (GO:0009117). The degradation of OTC by strain OTC-16 involves a series of redox reactions, including deheterocyclization and deorganocyclization, which align with the predicted gene functions.

To further explore the functions of the identified DEGs, KEGG pathway classification and enrichment analyses were performed. As shown in Fig. 2C and D, OTC significantly altered the metabolic behavior of strain OTC-16. Most DEGs were enriched in metabolic processes (aai01100) and microbial metabolic pathways in different environments (aai01120). We observed that the genes related to organic and heterocyclic compound binding in the GO enrichment analysis were primarily involved in these metabolic processes. This suggests that OTC exposure enhanced the strain’s binding to organic matter, which aligns with its degradation characteristics. Notably, the KEGG data revealed that most metabolic pathways were significantly upregulated, including glycine, serine, and threonine metabolism (aai00260), cysteine and methionine metabolism (aai00270), butyryl-CoA metabolism (aai00650), and propionate metabolism (aai00640). In contrast, the significantly downregulated pathways included pyrimidine and vitamin B6 metabolism (aai00240 and aai00750).

### Proteomics analysis of OTC biotransformation

Principal component analysis (PCA) and coefficient of variance (CV) analyses were performed on the protein data from strain OTC-16. The results indicated that the samples within the control and experimental groups clustered closely, and mixing was not observed between the groups (Fig. S3). Distinct separation among the treatment groups, minimal dispersion in the sample data, and good reproducibility confirmed the validity of the proteome sequencing data. A total of 352 DEPs were identified in the OTC-treated group on day 2 (*P* ≤ 0.05), including 230 upregulated proteins (fold change ≥1.2) and 122 downregulated proteins (fold change ≤0.83) compared with the CK group. A total of 818 DEPs were identified on day 4, including 418 upregulated and 400 downregulated proteins (Fig. 1C and D).

**Fig 3:**
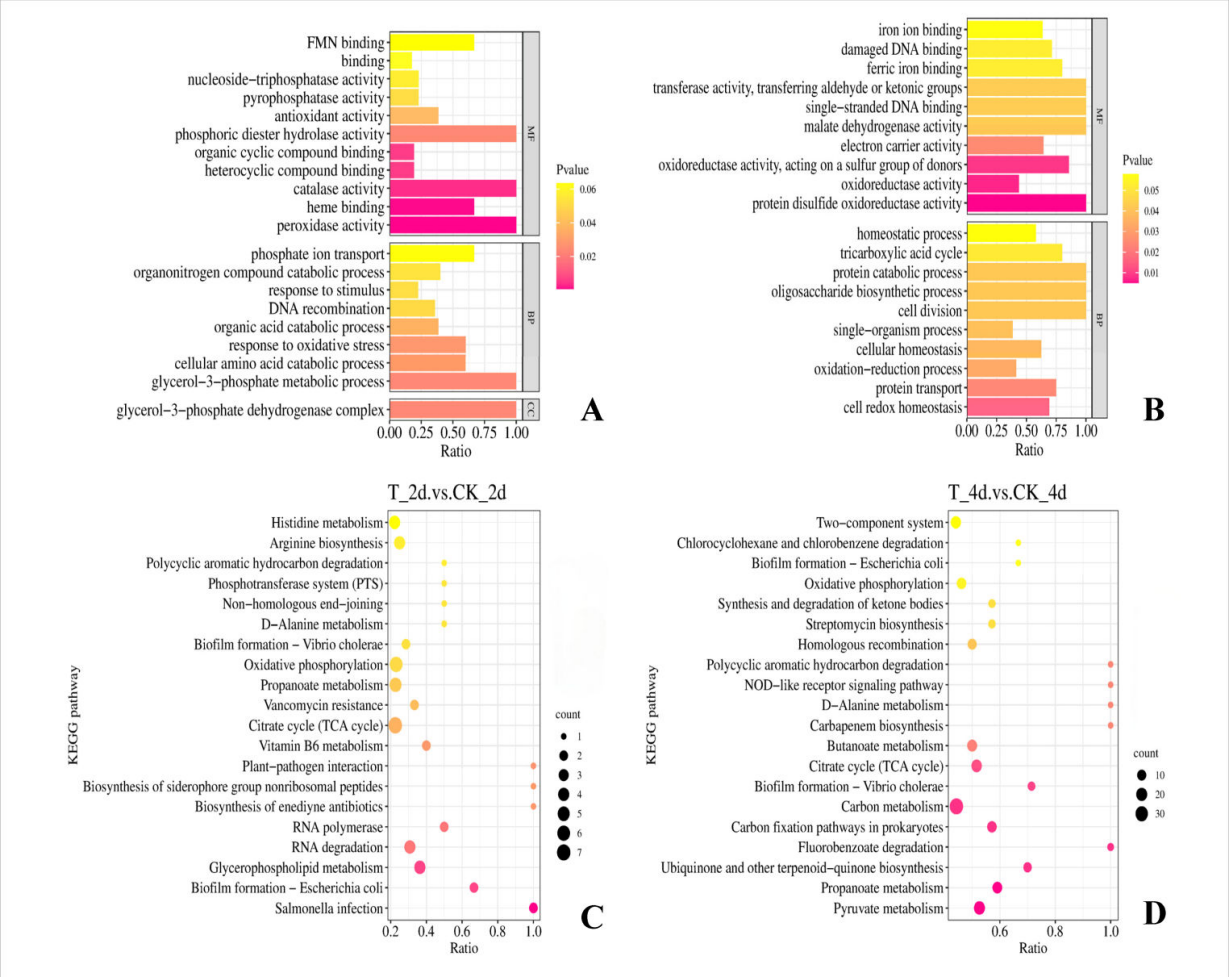
GO enrichment analysis circle plot and KEGG enrichment analysis bubble plot of DEPs of strain OTC-16 (**A**, **B**) GO enrichment analysis circle plot of DEPs on the 2nd and 4th days, respectively; (**C**,** D**) KEGG enrichment analysis bubble plot of DEPs on the 2nd and 4th days, respectively. In the GO enrichment analysis circle plot, the darker the color of the horizontal column, the more significant the difference. In the KEGG enrichment analysis bubble plot, the area of the dots is directly proportional to the enrichment degree of the metabolic pathways, with a larger area of the dots corresponding to a greater intensity of pathway enrichment.

GO enrichment analysis of DEPs in strain OTC-16 under OTC stress revealed that most DEPs were enriched in binding and redox processes on days 2 and 4 (Fig. 3). As shown in Fig. 3A, processes such as “binding” (GO:0005488), “organic cyclic compound binding” (GO:0097159), and “heterocyclic compound binding” (GO:1901363) were significantly enriched, consistent with the transcriptomic GO enrichment analysis results. Additionally, significant changes were observed in processes such as “peroxidase activity” (GO:0004601), “catalase activity” (GO:0004096), and “oxidative stress response” (GO:0006979). As shown in Fig. 3B, after the addition of OTC for 4 days, most DEPs were enriched in redox processes, including “oxidoreductase activity” (GO:0016667, GO:0016491), “protein disulfide oxidoreductase activity” (GO:0015035), “redox processes” (GO:0055114), and “cellular redox homeostasis” (GO:0045454). As most degradation enzymes have oxidoreductase activity, these redox processes are hypothesized to be involved in OTC degradation by this strain.

KEGG enrichment analysis revealed that most DEPs were primarily enriched in metabolic processes, which is consistent with the transcriptome enrichment analysis results. As shown in Fig. 3C, after 2 days of OTC exposure, significant enrichment was observed in pathways such as the TCA cycle (map00020), oxidative phosphorylation (map00190), propanoate metabolism (map00640), polycyclic aromatic hydrocarbon degradation (map00624), glycerophospholipid metabolism (map00564), and histidine metabolism (map00340). As shown in Fig. 3D, after 4 days of OTC exposure, most DEPs were enriched in the carbon metabolism pathway (map01200). Additionally, DEPs were enriched in biofilm formation (map02026) on both days 2 and 4.

### Identification of possible functional enzymes in OTC biotransformation by combined transcriptome and proteome analysis

**Fig 4:**
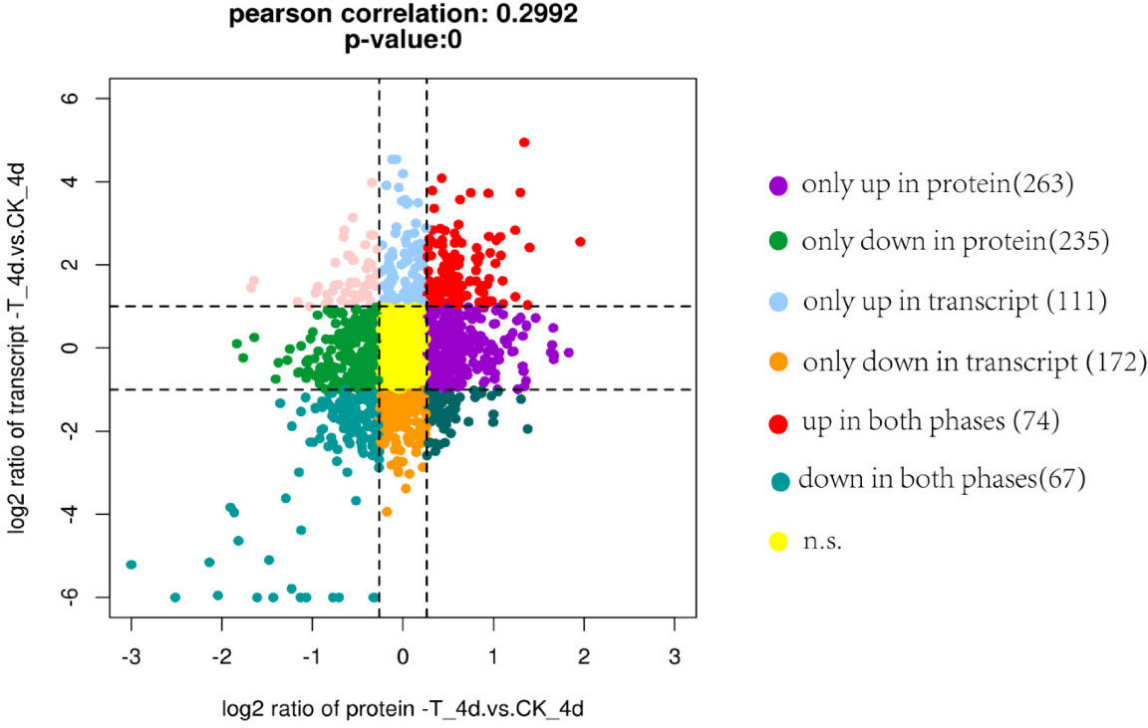
Transcriptome-proteome nine-quadrant plot of strain OTC-16 on day 4. Each dot corresponds to a gene (protein). The differentially expressed genes (proteins) are represented by different colors, which are marked in the picture.

Due to significant differences in protein expression levels on day 4, a joint analysis of the transcriptomic and proteomic sequencing data from day 4 of OTC treatment was performed, and a transcriptome-proteome nine-quadrant plot was drawn. As shown in Fig. 4, 74 genes exhibited coordinated upregulation at both the mRNA and protein levels, whereas 67 genes showed coordinated downregulation. KEGG pathway analysis of the genes with coordinated expression revealed that the genes upregulated at both the mRNA and protein levels were primarily enriched in metabolic pathways, such as “carbon metabolism” (aai01200), “pyrimidine metabolism” (aai00240), “propionate metabolism” (aai00640), and “amino acid biosynthesis” (aai01230). Downregulated genes were primarily enriched in pathways such as “ABC transporters” (aai02010) and “DNA replication” (aai03030).

**Fig 5:**
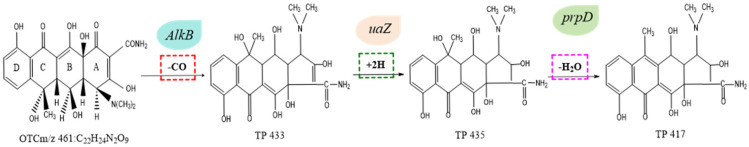
Biodegradation pathway and the proposed action site of the degrading gene in oxytetracycline biotransformation by strain OTC-16. The action site of genes *AlkB*, *prpD*, and *uaZ* was proposed based on a joint analysis of their decarbonylation, dehydration, and C = C bond reduction activity, as well as the known metabolic pathways of OTC-degradation by strain OTC-16 (8).

Annotation of the co-upregulated genes revealed two dehydrogenases, nine oxidases, and three reductases. Oxidoreductases, as core functional enzymes in the biodegradation of TC antibiotics, play a crucial role in antibiotic degradation. For example, flavin-dependent monooxygenases (30), laccase (31), lignin peroxidase (32), manganese peroxidase (33), and horseradish peroxidase (34) have strong capacities to degrade TC antibiotics. Therefore, we focused on analyzing oxidases and reductases in strain OTC-16’s biodegradation process. The genes *AlkB* (D3647_RS13630, encoding DNA oxidative demethylase [EC 1.14.11.33]), *prpD* (D3647_RS05790, encoding 2-methylcitrate dehydratase [EC 4.2.1.79]), and *uaZ* (D3647_RS16605, encoding urate oxidase [EC 1.7.3.3]) exhibited decarbonylation activity, dehydration activity, and C = C bond reduction activity, respectively, caught our attention. Based on a joint analysis of the known metabolic pathways underlying OTC degradation by strain OTC-16 (8) and the enzyme catalysis characteristics of these genes, they are hypothesized to be involved in the biodegradation of OTC by the strain. Their presumed action sites are shown in Fig. 5.

**Fig 6:**
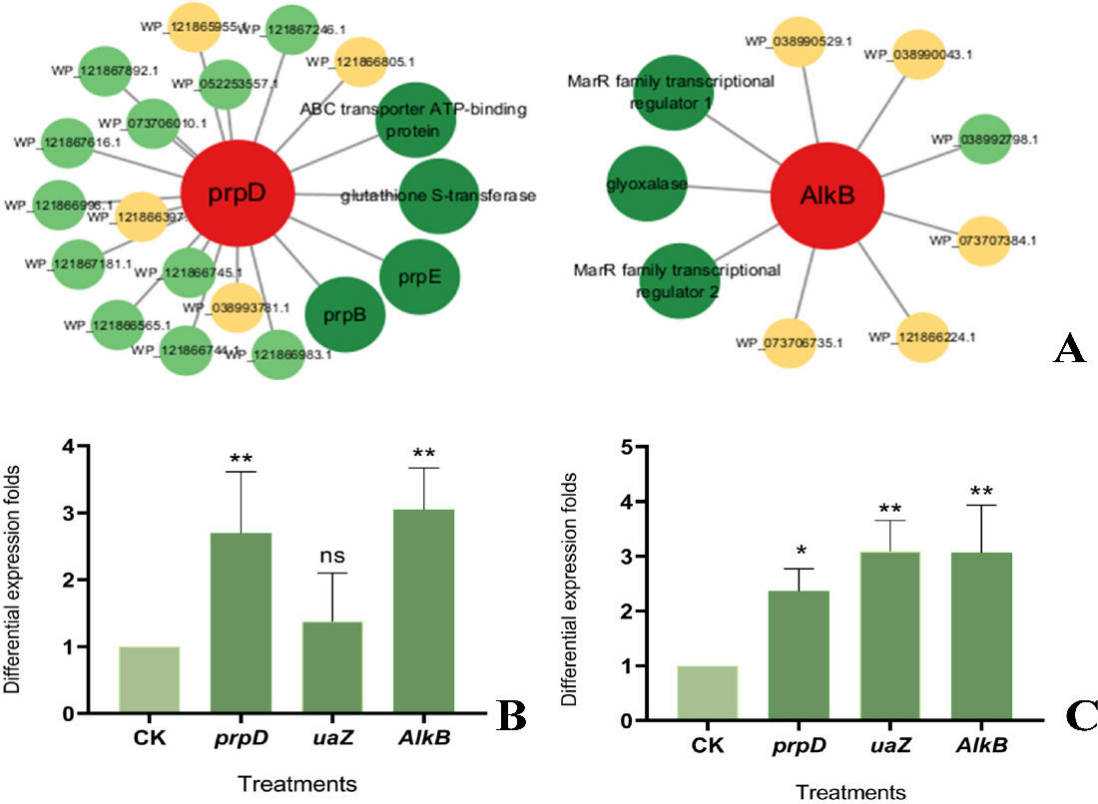
(**A**) PPI interaction analysis of the speculated degrading-related proteins (green represents upregulated genes, yellow represents downregulated genes, and darker colors indicate closer interactions with degraded proteins). (**B**) Expression levels of degradation enzyme genes on the 2nd day. (**C**) Expression levels of degradation enzyme genes on the 4th day.

PPI interaction analysis of the speculated degradation-related proteins revealed that most proteins positively interacted with the degrading genes (Fig. 6A). For example, 15 of 19 proteins positively regulated *prpD*. Among them, the glutathione S-transferase, ABC transporter ATP-binding, prpB, and prpE proteins had the maximum correlation coefficients, indicating that these proteins may have a greater influence on *prpD*. Of note, glutathione S-transferase has been reported to degrade TC antibiotics (35), whereas ABC transporter ATP-binding proteins are a class of transmembrane proteins capable of transporting multiple bioactive substances inside or outside the cell (36). These bioactive substances include antibiotics, toxins, nutrients, and metabolites. Therefore, these two proteins may play a significant role in OTC degradation by strain OTC-16. As shown in Fig. 6A, the MarR family of transcriptional regulators and glyoxalase are closely related to *AlkB*, and these proteins are suggested to play important roles in OTC degradation by interacting positively with *AlkB*. Notably, *prpD* and *AlkB* showed no direct interactions.

### Tracking OTC-induced gene expression of possible degrading enzymes by RT-qPCR

The relative expression of degradation genes identified via transcriptomic and proteomic analyses was assessed via RT-qPCR. The results demonstrated that *prpD*, *uaZ*, and *AlkB* were upregulated under OTC stress throughout the experiment, which indicates that these genes are induced by OTC and function in the OTC degradation process, as shown in Fig. 6B and C. The consistent upregulation patterns of these three predicted degradation genes with the transcriptomic data further validate their reliability.

Although all genes were upregulated, the degree of upregulation varied. Only *prpD* and *AlkB* showed significant upregulation on day 2, whereas all three genes were significantly upregulated on day 4, indicating that the three genes were expressed sequentially rather than simultaneously.

### Functional studies for possible OTC biodegradation enzymes

**Fig 7:**
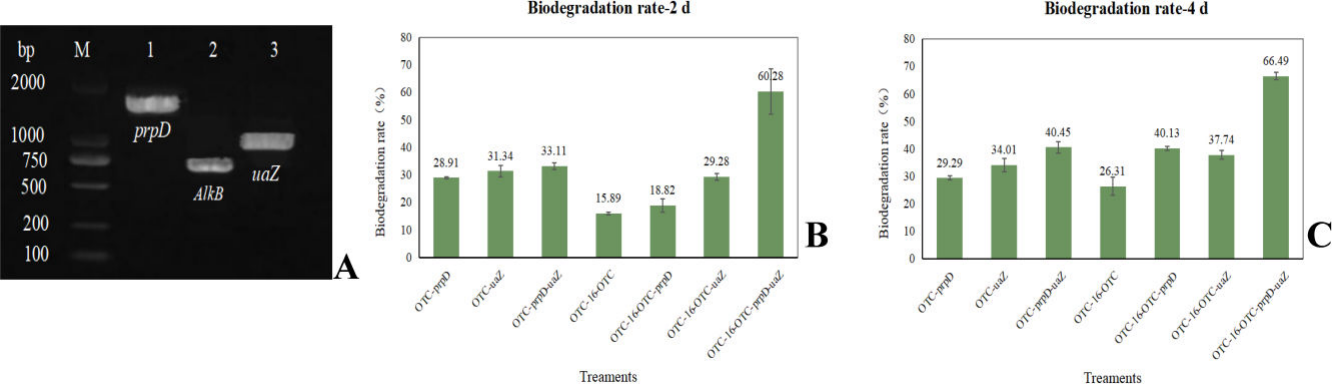
(**A**) Electrophoretic map of genes *prpD*, *AlkB,* and *uaZ* (M: DNA marker; 1, 2, and 3: PCR products of genes *prpD*, *AlkB*, and *uaZ*, respectively). (**B**) Degradation efficiency of oxytetracycline in each treatment on the 2nd day. (**C**) Degradation efficiency of oxytetracycline in each treatment on the 4th day.

PCR amplification of the degrading gene was performed, and the amplified products were analyzed using 1% agarose gel electrophoresis. Three amplified fragment segments of 693 bp, 947 bp, and 1541 bp corresponded to the expected sequences of *AlkB, uaZ*, and *prpD*, respectively (Fig. 7A). The gene fragments were then ligated into plasmid pET-28a (+) through enzymatic digestion and ligation to construct the recombinant plasmids pET-28a (+)-*AlkB*, pET-28a (+)-*uaZ*, and pET-28a (+)-*prpD*. The resulting plasmids were transformed into *E. coli* DH5α for plasmid amplification. The constructed recombinant pET-28a (+) expression plasmid was extracted and sequenced by Qingke Biotech (Beijing, China) to confirm the absence of mutations in the inserted target fragment. Sequence alignment confirmed the successful construction of all three recombinant plasmids (Fig. S1B through D).

**Fig 8:**
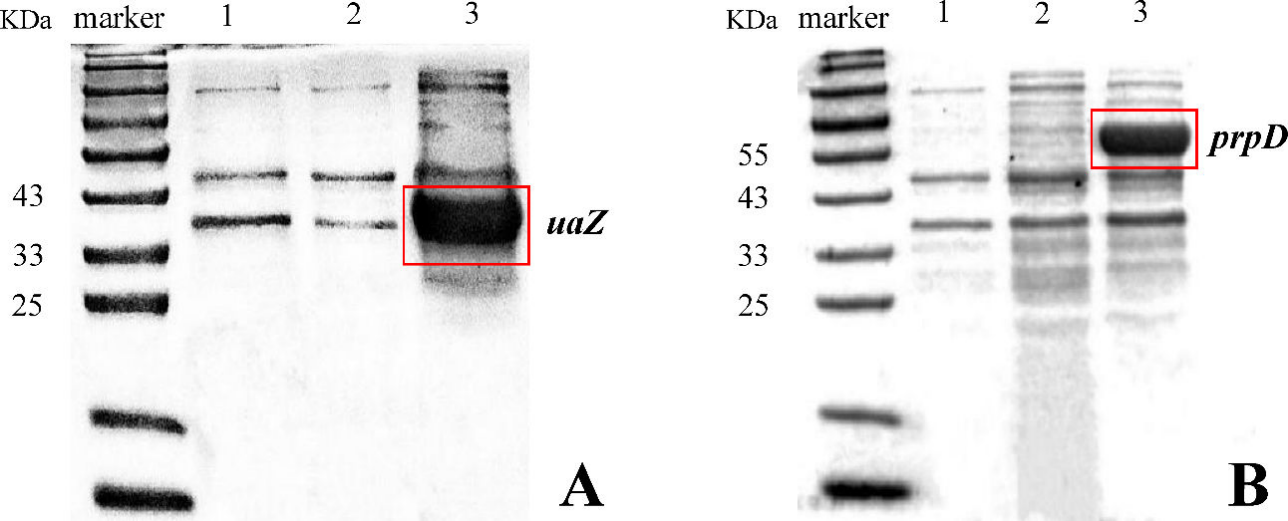
Protein electrophoresis analysis of the induced expression of degrading genes *uaZ* (**A**) and *prpD* (**B**). Marker indicates protein molecular weight standards; 1: Supernatant of the cell lysate of the strain containing pET-28a (+). 2: Supernatant of the uninduced cell lysate of the strain containing recombinant plasmids. 3: Induced supernatant of the cell lysate of the strain containing recombinant plasmids.

Positive transformants carrying the target genes were induced with IPTG for protein expression, which was analyzed by 12% SDS-PAGE (Fig. 8). The electrophoresis lanes of *prpD* and *uaZ* showed two protein bands with molecular weights of 55 and 34 KDa, respectively, matching their theoretical relative molecular weights. Both proteins were primarily present in a soluble manner in the supernatant, indicating their successful expression in *E. coli*. However, the recombinant *AlkB* strain failed to express the AlkB protein in the electrophoresis lane, suggesting that the AlkB protein may not be efficiently expressed in *E. coli* in a soluble manner. This assumption is supported by research by Cui (29), which showed that AlkB exists as an insoluble inclusion body in *E. coli*. Cui speculated that inclusion body formation might result from IPTG-induced overexpression of *AlkB*, exceeding normal expression levels.

To verify the degradation potential of expression products of gene *prpD* and *uaZ*, residual OTC levels in different systems were measured after 2 and 4 days of incubation. As illustrated in Fig. 7B and C, the degradation proteins prpD and uaZ exhibited degradation effects on OTC, regardless of the presence of strain OTC-16. Moreover, their combined use resulted in higher degradation rates than individual treatments. For instance, on day 4, OTC degradation reached 40.45% when both proteins were used together, whereas it reached values of 29.29% and 34.01% when *prpD* and *uaZ* were used alone, respectively. Additionally, the expressed proteins enhanced OTC degradation by strain OTC-16. On day 2, OTC degradation by strain OTC-16 alone was 15.89%, whereas on day 4, it increased to 26.31%. In contrast, in treatments where strain OTC-16 was combined with both degradation proteins, OTC degradation reached 60.28% and 66.49% on days 2 and 4, respectively.

Although *AlkB* has been reported to be a key gene in the degradation of diesel and petroleum alkanes (37, 38), none of these three genes has been previously associated with antibiotic degradation. This study is the first to confirm that *prpD* and *uaZ* play pivotal roles in OTC residue removal. These findings have significantly advanced our understanding of the biodegradation mechanism of OTC.

### Conclusion

This study integrated transcriptomic and proteomic analyses to elucidate the molecular mechanisms underlying OTC biodegradation by *Arthrobacter nicotianae* OTC-16. Significant gene expression changes occurred upon OTC exposure, primarily influencing compound binding and oxidoreduction processes. AlkB, uaZ, and prpD were identified as pivotal enzymes facilitating OTC degradation, supported by interactions with glutathione S-transferase, ABC transporter ATP-binding protein, prpB, prpE, MarR transcriptional regulators, and glyoxalase. Experimental validation via RT-qPCR and recombinant protein expression confirmed the roles of *prpD* and *uaZ*, marking their first reported involvement in antibiotic degradation. These findings deepen our understanding of OTC degradation mechanisms and highlight promising targets for biotechnological enhancement and environmental applications.

## Data Availability

All analysis tools used in the study are publicly available. The original data were deposited in the NCBI database (https://www.ncbi.nlm.nih.gov/bioproject/) and the accession is PRJNA490584.

## SUPPLEMENTAL MATERIAL

Supplemental Material spectrum.00562-25-s0001.docx Table S1; Fig. S1 to S3.

ASM does not own the copyrights to Supplemental Material that may be linked to, or accessed through, an article. The authors have granted ASM a non-exclusive, world-wide license to publish the Supplemental Material files. Please contact the corresponding author directly for reuse.
