# Supplementary material for: Novel enzymes involved in the biotransformation of oxytetracycline by Arthrobacter nicotianae OTC-16
Source: Microbiol Spectr. 2025 Aug 12;13(9):e00562-25. doi: 10.1128/spectrum.00562-25 (PMC12403558; doi:10.1128/spectrum.00562-25)
Supplement: Supplemental Material — Table S1; Fig. S1 to S3. [file spectrum.00562-25-s0001.docx]

**Novel enzymes involved in the biotransformation of oxytetracycline by *Arthrobacter nicotianae* OTC-16**

Beibei Wang^1^, Weijie Xu^1^, Keke Wang^1^, Hui Lin^2*^, Xin Zhang^1*^, Zulfiqar Ahmad^3^

^1^ *College of Forest and Biotechnology, Zhejiang A & F University, Hangzhou 311300, China*

*^2^ Institute of Environment, Resource, Soil and Fertilizer, Zhejiang Academy of Agricultural Sciences, Hangzhou, China*

^3^*Laboratoire Ondes et Milieux complexes (LOMC), UMR 6294 CNRS, University of Le Havre Normandy (ULHN), 76600, Le Havre, France*

**^*^ Corresponding authors at:** College of Forest and Biotechnology, Zhejiang A & F University, E-mail address: zhangxins@126.com (X. Zhang); The Institute of Environment, Resources, Soil and Fertilizers, Zhejiang Academy of Agricultural Sciences, Tel: +86-571-86404302, Fax: +86-571-86404302, E-mail address: linhui@zaas.ac.cn (H. Lin)

**Captions**

**Table S1** Quality statistics of RNA sequencing data. Note: A1, B1, and C1 represent the three biological replicates of OTC-16 cultured with OTC exposure for 2 days, respectively; a-1, b-1, and c-1 represent the three biological replicates of OTC-16 cultured for 2 days without OTC; A2, B2, and C2 represent the three biological replicates of OTC-16 cultured with OTC for 4 days, respectively. a-2, b-2, and c-2 represent the three biological replicates of OTC-16 cultured for 4 days without OTC.

**Figure S1** (A) Schematic diagram of pET-28a(+) plasmid; (B) Schematic diagram of recombinant plasmid pET-28a(+)-*prpD*; (C) Schematic diagram of recombinant plasmid pET-28a(+)-*AlkB*; (D) Schematic diagram of recombinant plasmid pET-28a(+)-*uaZ*.

**Figure S2** Transcriptome-related heat map. Note: A1, B1, and C1 represent the three biological replicates of OTC-16 cultured with OTC exposure for 2 days, respectively; a-1, b-1, and c-1 represent the three biological replicates of OTC-16 cultured for 2 days without OTC; A2, B2, and C2 represent the three biological replicates of OTC-16 cultured with OTC for 4 days, respectively. a-2, b-2, and c-2 represent the three biological replicates of OTC-16 cultured for 4 days without OTC.

#### **Figure S3** Principal Component Analysis (A) and repeated Coefficient of Variance (B) analysis of differentially expressed protein.

**Table S1** Quality statistics of RNA sequencing data


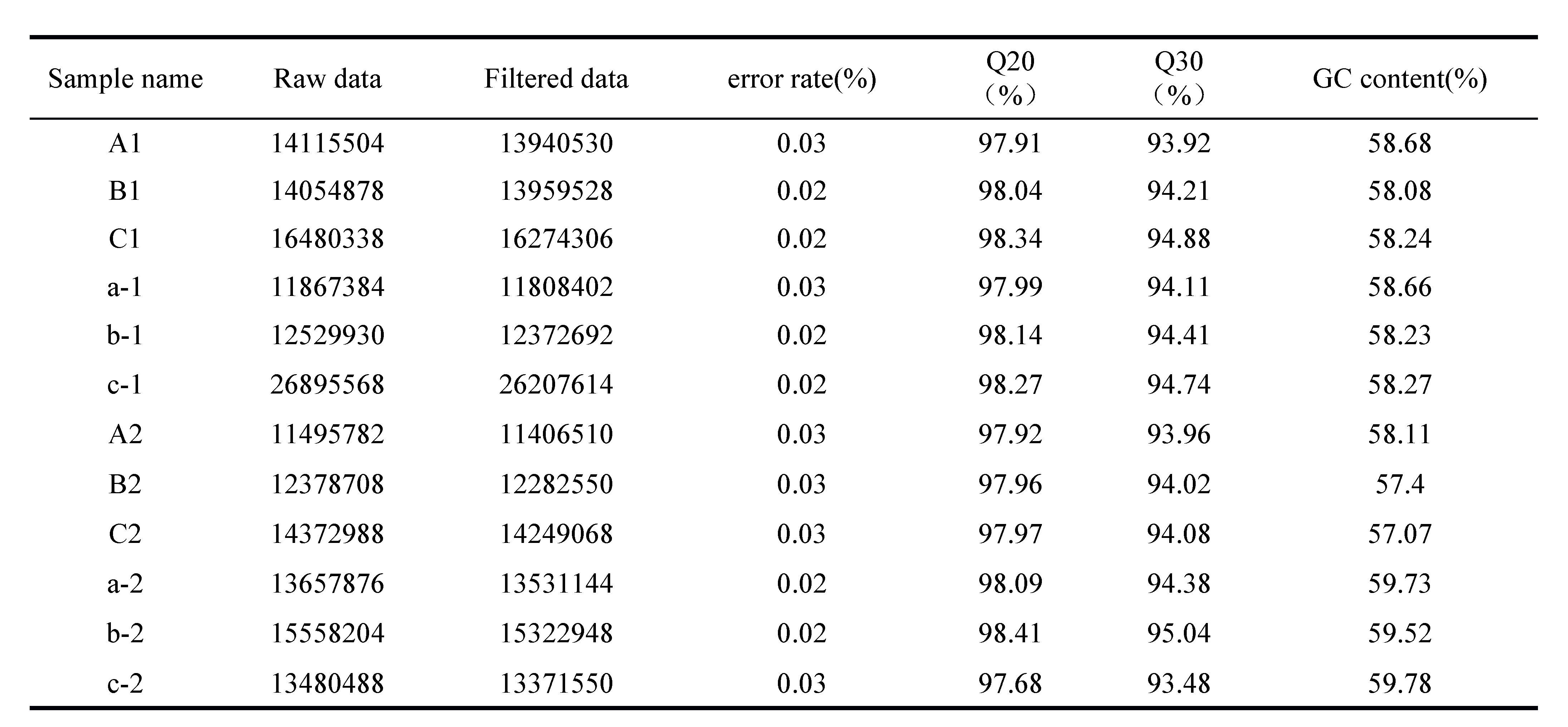


Note: A1, B1, and C1 represent the three biological replicates of OTC-16 cultured with OTC exposure for 2 days, respectively; a-1, b-1, and c-1 represent the three biological replicates of OTC-16 cultured for 2 days without OTC; A2, B2, and C2 represent the three biological replicates of OTC-16 cultured with OTC for 4 days, respectively. a-2, b-2, and c-2 represent the three biological replicates of OTC-16 cultured for 4 days without OTC. Q20: The percentage of bases with mass values greater than 20 (error rate less than 1%) to the total bases; Q30: The percentage of bases with mass values greater than 30 (error rate below 0.1%) out of the total bases.


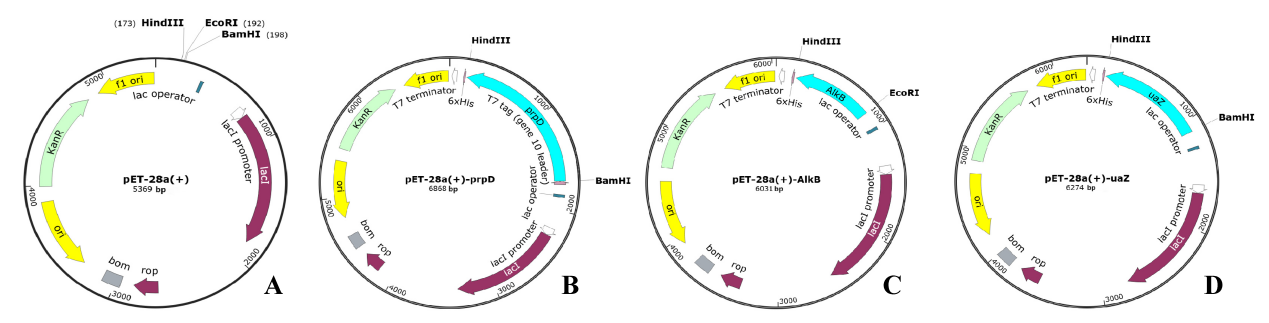


**Figure S1** (A) Schematic diagram of pET-28a(+) plasmid; (B) Schematic diagram of recombinant plasmid pET-28a(+)-*prpD*; (C) Schematic diagram of recombinant plasmid pET-28a(+)-*AlkB*; (D) Schematic diagram of recombinant plasmid pET-28a(+)-*uaZ.*


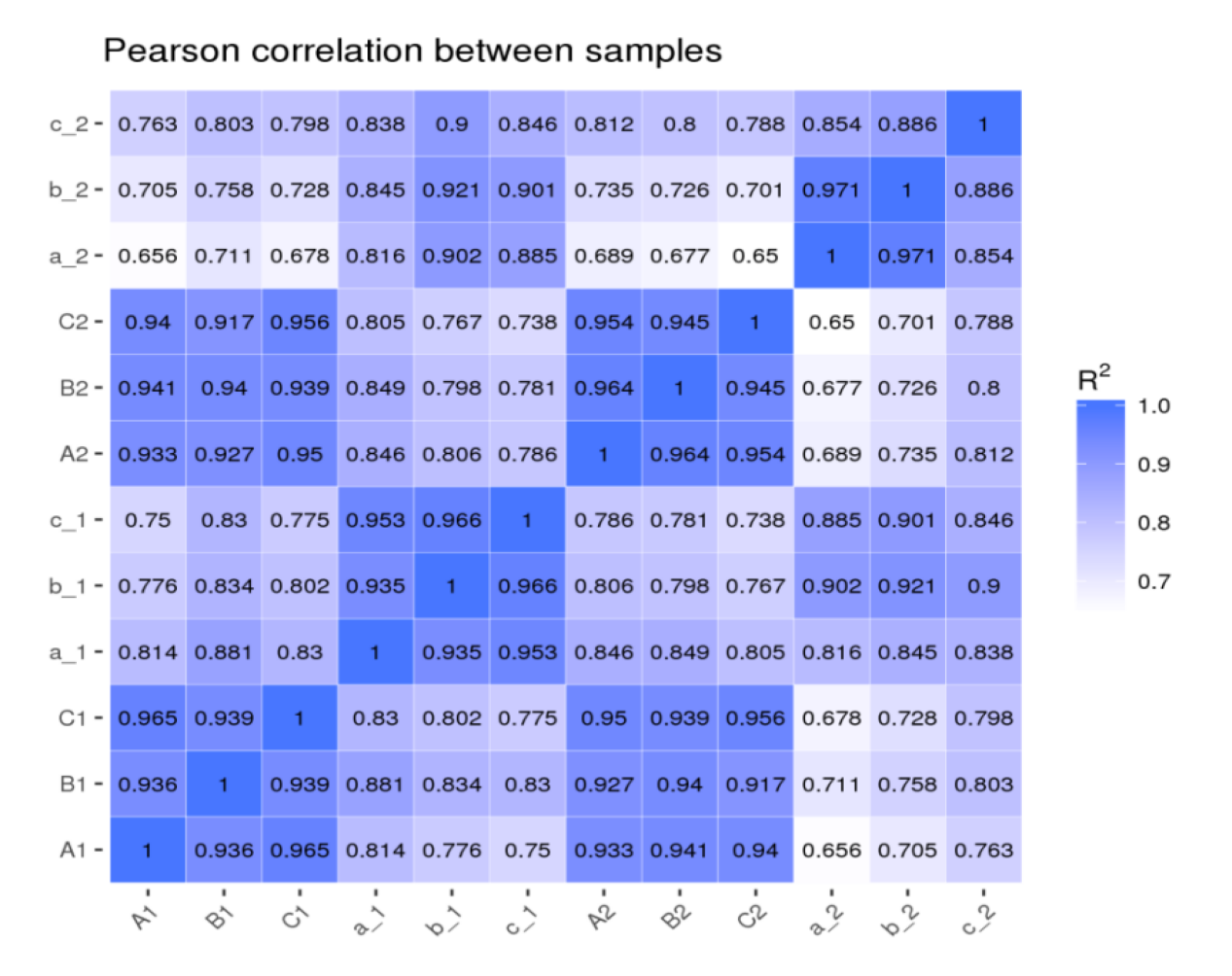


**Figure S2** Transcriptome-related heat map

Note: A1, B1, and C1 represent the three biological replicates of OTC-16 cultured with OTC exposure for 2 days, respectively; a-1, b-1, and c-1 represent the three biological replicates of OTC-16 cultured for 2 days without OTC; A2, B2, and C2 represent the three biological replicates of OTC-16 cultured with OTC for 4 days, respectively. a-2, b-2, and c-2 represent the three biological replicates of OTC-16 cultured for 4 days without OTC.


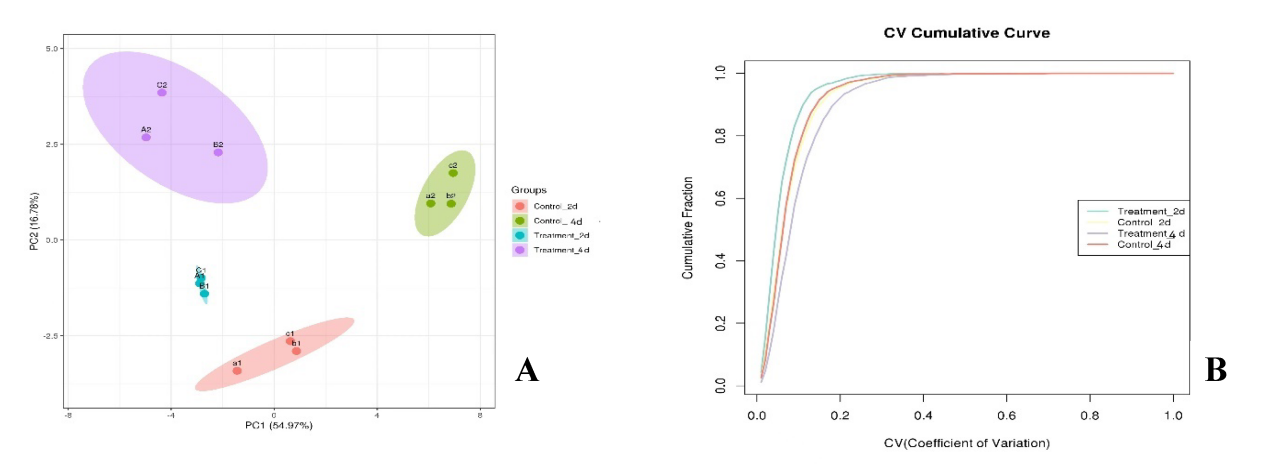
**Figure S3** Principal Component Analysis (A) and repeated Coefficient of Variance (B) analysis of differentially expressed protein.
